# Direct bioconversion of brown algae into ethanol by thermophilic bacterium *Defluviitalea phaphyphila*

**DOI:** 10.1186/s13068-016-0494-1

**Published:** 2016-04-01

**Authors:** Shi-Qi Ji, Bing Wang, Ming Lu, Fu-Li Li

**Affiliations:** Key Laboratory of Biofuels, Shandong Provincial Key Laboratory of Synthetic Biology, Qingdao Institute of Bioenergy and Bioprocess Technology, Chinese Academy of Sciences, 189 Songling Road, Qingdao, 266101 People’s Republic of China

**Keywords:** Brown algae, Redox balance, Bioethanol, Marine thermophile, *Defluviitalea phaphyphila*

## Abstract

**Background:**

Brown algae are promising feedstocks for biofuel production with inherent advantages of no structural lignin, high growth rate, and no competition for land and fresh water. However, it is difficult for one microorganism to convert all components of brown algae with different oxidoreduction potentials to ethanol. *Defluviitalea phaphyphila* Alg1 is the first characterized thermophilic bacterium capable of direct utilization of brown algae.

**Results:**

*Defluviitalea phaphyphila* Alg1 can simultaneously utilize mannitol, glucose, and alginate to produce ethanol, and high ethanol yields of 0.47 g/g-mannitol, 0.44 g/g-glucose, and 0.3 g/g-alginate were obtained. A rational redox balance system under obligate anaerobic condition in fermenting brown algae was revealed in *D. phaphyphila* Alg1 through genome and redox analysis. The excess reducing equivalents produced from mannitol metabolism were equilibrated by oxidizing forces from alginate assimilation. Furthermore, *D. phaphyphila* Alg1 can directly utilize unpretreated kelp powder, and 10 g/L of ethanol was accumulated within 72 h with an ethanol yield of 0.25 g/g-kelp. Microscopic observation further demonstrated the deconstruction process of brown algae cell by *D. phaphyphila* Alg1.

**Conclusions:**

The integrated biomass deconstruction system of *D. phaphyphila* Alg1, as well as its high ethanol yield, provided us an excellent alternative for brown algae bioconversion at elevated temperature.

## Background

To settle worldwide energy crisis and environmental problems, increased interests have been focused on aquatic and marine production for biofuels, which mainly includes biofuels derived from macroalgae and microalgae. Brown algae are a large group of marine seaweeds including almost 1800 species of macroalgae with a characteristic olive-green to dark brown color derived from fucoxanthin [1]. Some well-known aquaculture or wild species are from the genera of *Laminaria*, *Saccharina*, *Macrocystis*, *Sargassum,* and so on. The technology for the mass production of macroalgae has been developed significantly in China and Asia over the last 50 years [2]. Notably, China contributes 72 % of global aquaculture-based macroalgae production, including the genera of *Laminaria* (reclassified as *Saccharina* for some species, brown algae), *Undaria* (green algae), *Porphyra*, and *Gracilaria* (red algae) [3]. Brown algae have complex sugar composition, mainly including alginate, mannitol, and laminarin [3]. Alginate is the unique structural polysaccharides in brown algae, which consists of two uronic acids, namely, α-l-guluronate (G) and β-d-mannuronate (M) [4]. The content of alginate varied from 20 to 40 % of dry weight among different species [5, 6]. Mannitol and laminarin are considered as reserve carbohydrates in many brown algae species, which are mostly accumulated in summer. Mannitol is a sugar alcohol form of mannose, while laminarin is a linear polysaccharide of mannitol-containing β-1,3-linked glucose [7, 8]. The content of mannitol and laminarin in some species can reach as high as 25 and 30 %, respectively, at the beginning of autumn [9].

The inherent advantages of brown algae for biofuel production mainly include the structural advantage of containing no lignin, high growth rate, and no competition with food production for land or fresh water [1, 10, 11]. They have been used for anaerobic digestion to produce biogas and liquid biofuel production. The direct bioconversion of brown algae to produce bioethanol cannot be easily achieved because of their diverse carbohydrate components. It is difficult for one microorganism to ferment all saccharides for biofuel production. Although glucose released from the hydrolysis of glucan could be easily assimilated through glycolysis by candidate strains, mannitol catabolism needs additional enzymes before entering glycolysis which include d-mannitol phosphotransferase (PTS) permease which transports d-mannitol into cells with the formation of mannitol-1-phosphate, and one mannitol-1-phosphate dehydrogenase (MPDH) (mannitol degradation I, MetaCyc Pathway Database, http://www.metacyc.org/) [12]. One reduced nicotinamide adenine dinucleotide (NADH) or reduced nicotinamide adenine dinucleotide phosphate (NADPH) was produced in this process of oxidizing mannitol-1-phosphate to fructose-6-phosphate. Moreover, the saccharification of brown algae requires one microorganism to secrete several polysaccharide depolymerizing enzymes such as alginate lyase and laminarinase. Through the endolytical and exolytical cleavages by alginate lyase and oligoalginate lyases, alginate was degraded into unsaturated monosaccharide (spontaneously rearranged into 4-deoxy-L-erythro-5-hexoseulose uronic acid, DEH) [13]. Subsequently, a DEH reductase and one 2-keto-3-deoxy-d-gluconate (KDG) kinase converted DEH into 2-keto-3-deoxy-6-phosphogluconate (KDPG) with a consumption of one NADH or NADPH, and then KDPG was directly assimilated through the Entner–Doudoroff (ED) pathway [14, 15]. In general, most ethanologenic microorganisms did not contain these genes encoding alginate depolymerizing enzymes. Thus, acid or enzymatic pretreatments were needed to decompose their structural polysaccharides to release monomer sugars from brown algae biomass [11, 16–19]. Moreover, the combined metabolism of alginate and mannitol also needs a well evolved redox system to balance the reducing equivalents, especially under anaerobic fermentation conditions [20]. Thus, in a direct bioconversion process for ethanol production, one microorganism need to secrete multiple enzymes to depolymerize polysaccharides, uptake the released sugars, metabolize the sugars, and balance the redox state of the cells. Due to these limitations, only a few natural microorganisms exhibit all the features desired for direct bioconversion of brown algae as far as we know. However, several natural strains showing partial desirable properties have been reported [17, 21, 22]. In general, they could only utilize glucan and/or mannitol released from brown algae after acid or enzyme pretreatment for bioethanol production. To generate a viable organism with better performance in brown algae bioconversion, attempts to genetically engineer natural strains were reported. For example, *Escherichia coli* was engineered to a microbial platform for bioethanol production directly from brown macroalgae by introducing a DNA fragment from *Vibrio splendidus* encoding alginate transport and metabolism and ethanol synthesis genes (*pdc* and *adhB*) from *Zymomonas mobilis* [23]. Recently, a synthetic yeast platform (*S. cerevisiae*) has been constructed to be able to metabolize DEH and mannitol, however, without alginate degradation ability [20]. The procedure of genetic engineering is extremely complex, and besides it is hard to meet all the requirements which favored direct fermentation processes.

In our previous work, an untapped thermophilic bacterial resource was identified from coastal sediments [24–26]. Strain *Defluviitalea phaphyphila* Alg1 is one of the species isolated from this environment [27]. Genome analysis indicated that strain Alg1 has an integrated brown algae-degrading system. In this work, the potential of *D. phaphyphila* Alg1 in direct bioconversion of brown algae to ethanol was investigated and evaluated.

## Methods

### Culture media and microorganisms

*S. japonica* was purchased from Tuandao seafood market in Qingdao, China. The seaweed was dried under sunlight and then ground into powder by a knife mill. The powder was filtered through a 200-mesh sieve. The basal medium (BM) consisted of 0.1 g/L of KH_2_PO4, 0.1 g/L of K_2_HPO4, 1 g/L of NaHCO_3_, 2 g/L of NH_4_Cl, 30 g/L of sea salt, 0.5 g/L of l-cysteine, 0.2 % of yeast extract, and 0.0001 % (w/v) of resazurin. The BM medium containing 1 g/L of alginate (ABM) was sterilized at 115 °C for 20 min and used as inoculum medium. The previously characterized type strain *D. phaphyphila* Alg1 (= CGMCC 1.5199^T^ = JCM 30481^T^) was used as fermentation strain [27]. A seed culture (50 ml ABM medium in anaerobic bottle) was prepared from a single colony of Alg1 from plate and incubated at 60 °C for 24 h until the optical density at 600 nm (OD_600_) reached 0.8–0.9. For fermentation experiments, a culture was inoculated with 5 % seed culture (v/v).

### Ethanol fermentation of *D. phaphyphila* Alg1

Fermentations were carried out in 250 ml bottle containing 100 ml BM at 60 °C and obligate anaerobic conditions by supplementing 1 % (w/v) of each substrate including glucose, mannitol, and alginate, respectively. A mixture of alginate, mannitol, and glucose (at a ratio of 5:8:1) with 3 % total sugar was used to simulate the main components of brown algae [23], and was tested in the same way as above. Fermentation of *S. japonica* powder was carried out in 1-L bioreactors. The fermentation medium consisted of 0.5 % yeast extract and 5 % milled kelp powder in BM. A constant flow of pure nitrogen was bubbled into the medium to remove dissolved oxygen and to maintain anaerobic conditions through the whole fermentation process. The fermentation was carried out under static conditions without stirring. Preliminary experiments have suggested that mechanical mixing was not favored by *D. phaphyphila* Alg1 in kelp fermentation. Samples were taken periodically, and were frozen at −20 °C until further analysis. All experiments were carried out in triplicate.

### Genome analysis of ethanol synthesis pathway and redox balance analysis

The Whole Genome Shotgun project of *D. phaphyphila* Alg1 has been deposited at DDBJ/EMBL/GenBank under the accession number of JWID00000000. The version described in this paper is version JWID01000000.

The redox balance of anaerobic metabolism of glucose, mannitol, alginate, and their mixture was analyzed as follows. The cells growing in different substrates were collected in late exponential phase. An Amplite™ Colorimetric NAD/NADH Ratio Assay Kit (AAT Bioquest Inc., Sunnyvale, CA, USA) was used to measure the intracellular total NAD/NADH amount and NAD/NADH ratio in culture cells according to the manual. Briefly, NAD in the cell lysate was extracted with NAD extraction solution and converted to NADH through enzyme reaction, and then recognized by the NADH probe to give a yellow-color dye after reaction, which has the absorbance at 460 nm. The amount of the dye generated is directly proportional to the concentration of NAD or NADH in the cell lysate.

### Microscopic observation of cell wall morphology change

The morphological changes of brown algae during fermentation were examined using optical microscope (Olympus BX51, Tokyo, Japan) and transmission electron microscope (TEM) (Hitachi H-7650, Tokyo, Japan). Briefly, brown algae were cut into 2 cm in width and 5 cm in length, and were added into the fermentation bottle. After 120 h of fermentation, the remaining brown algae specimens were cut into 0.1–0.2 cm pieces. For optical microscopy, brown algae pieces were stained by 0.1 % crystal violet for 10 min and rinsed two times with phosphate buffer. For transmission electron microscopy, cells were fixed by 2.5 % glutaraldehyde solution and 1 % osmic acid. Tissue was dehydrated in graded acetone starting with 30, 50, 70, 80, 90, 95 % for 30 min each and with 3 rinses in 100 % acetone. Infiltrate with acetone/Spurr resin (7:3) for 5 h, acetone/Spurr (3:7) overnight and 100 % Spurr resin for 5 h. Embed tissue in fresh Spurr resin and place in 65 °C oven and polymerize for 24 h. Leica EM UC6 (Vienna, Austria) was used to prepare ultrathin sections (70 nm) to examine the cell ultrastructure, and uranyl acetate (2 %) was used to stain the sections.

### Analytical methods for cell growth, products, and enzyme activities

Cell growth was monitored by measuring OD_600_ using a lambda 25 UV/Vis spectrometer (PerkinElmer, Shelton, USA). In the case of alginate as substrate, solid particles in the medium prevented the determination of cell density with the spectral photometer, therefore, the cell growth was assessed by measuring the total protein concentrations using the Bradford assay kit (Biomed, Beijing, China) with bovine serum albumin as the standard [28]. The concentrations of mannitol, glucose, and fermentation products were determined using high-performance liquid chromatography (HPLC) equipped with an Aminex HPX-87H column (Bio-Rad, Hercules, USA). Filtered and degassed H_2_SO_4_ (5 mM) was used as eluent at a flow rate of 0.6 ml/min and the column temperature was controlled at 65 °C. Quantification of alginate was performed using the carbazole sulfuric acid method with sodium alginate as the standard [29].

The alginate lyase and laminarinase activities were determined during the fermentation of kelp powder. Alginate lyase activity was assayed by measuring the increase in absorbance at 235 nm (A_235_) of the reaction products (unsaturated uronates) for 3 min at 70 °C in a quartz cuvette containing 2 ml of 0.2 % alginate in 100 mM acetate/sodium acetate buffer (pH 5.8) and 10 µl fermentation supernatant. One unit of activity was defined as an increase of 0.1 in A_235_ per minute. Laminarinase activity was assayed by incubating laminarin solution (1 % w/v, in 50 mM Tris/HCl, pH 7.3) with fermentation supernatant at 70 °C for 3 min. The release of reducing sugars was measured by the dinitrosalicylic (DNS) colorimetric method [30]. One unit of laminarinase activity was defined as 1 mg of reducing sugar equivalent to 1 mg of glucose per minute.

## Results and discussion

### Fermentation of glucose, mannitol, and alginate for ethanol production

**Fig. 1:**
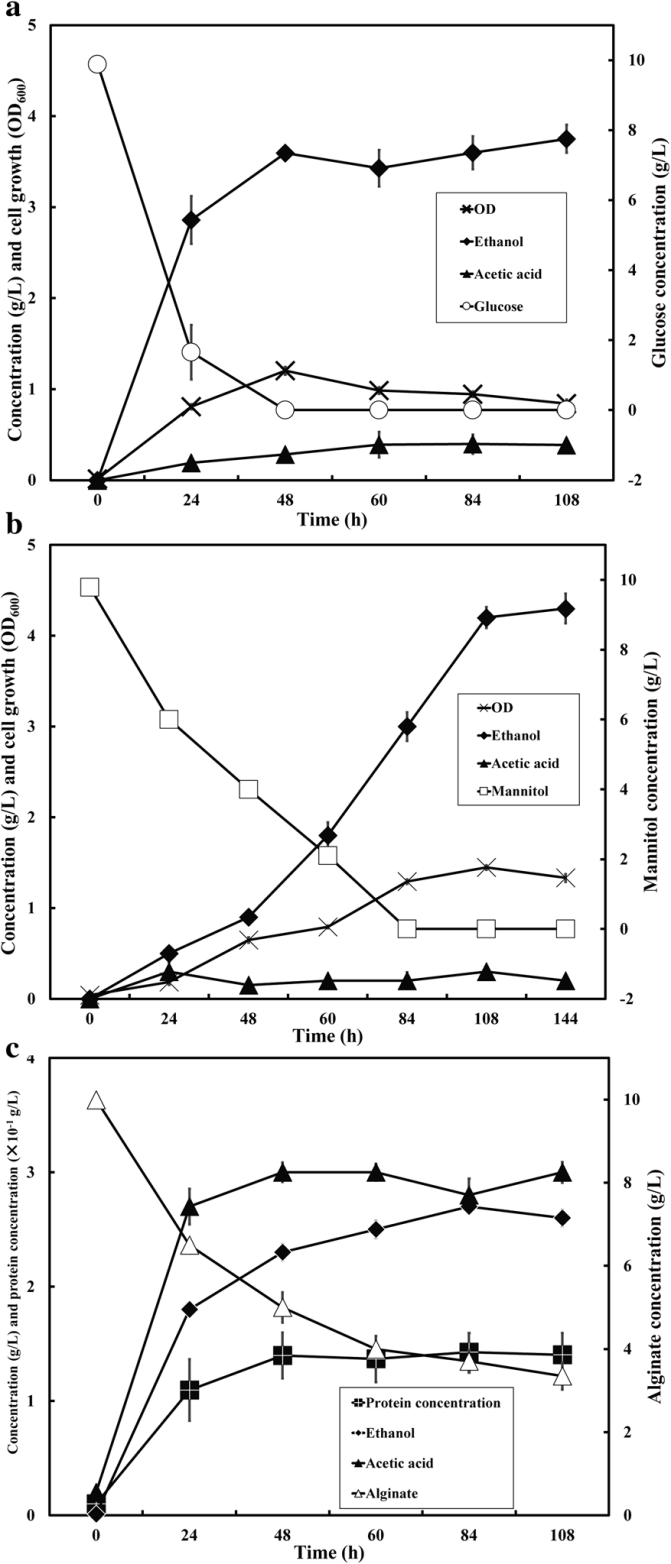
Microbial growth, substrate consumption, and ethanol production of *D. phaphyphila* Alg1 with the substrates of glucose (1 % w/v, **a**), mannitol (1 % w/v, **b**), and alginate (1 % w/v, **c**)

The growth and ethanol fermentation properties of *D. phaphyphila* Alg1 were evaluated by employing alginate, mannitol, or glucose (laminarin monomer) as substrates. When glucose (1 %, w/v) was used as sole carbon substrate, Alg1 showed vigorous growth after inoculation, and all glucose was exhausted in less than 48 h. The maximal cell concentration (OD_600_) reached 1.2 at 48 h (Fig. 1a). After 108 h cultivation, 3.8 g/L ethanol and 0.4 g/L acetic acid were produced, with an ethanol-to-acetate ratio of 9.5:1 (Fig. 1a). The ethanol yield was 0.38 g/g-glucose which accounted for 74 % of the maximal yield of 0.51 g/g-glucose [31]. In the case of mannitol (1 %, w/v) as substrate, the cell density could reach to the highest OD_600_ of 1.4 after 108 h cultivation (Fig. 1b). The concentrations of ethanol and acetic acid could be as high as 4.3 and 0.3 g/L, respectively, with an ethanol-to-acetate ratio around 14:1 (Fig. 1b). The ethanol yield from mannitol was about 0.44 g/g-mannitol, which accounted for 86 % of the theoretical maximal yield of 0.51 g/g-mannitol [8]. Mannitol was exhausted until 108 h indicating a slower substrate assimilation rate than glucose. Indeed, from the reported literatures, mannitol normally cannot be fermented under strictly anaerobic condition by *Zymobacter palmae*, *Enterobacter* sp. JMP3 and some yeast species, while glucose can be easily converted to ethanol under the same condition. Thus, the utilization of mannitol is more complex, and has higher requirements on microorganisms. In addition, in contrast to those of *Z. palmae* (0.38 g-ethanol/g-mannitol) and *Enterobacter* sp. (0.29 g-ethanol/g-mannitol) [8, 22], *D. phaphyphila* Alg1 showed the highest ethanol conversion rate of 0.44 g-ethanol/g-mannitol, which suggests Alg1 is a potential strain for mannitol bioconversion. Alginate could not be fully dissolved in the medium for the gelling property in the presence of divalent ion. Thus, total protein concentration of cells was used to monitor the growth of *D. phaphyphila* Alg1 in the case of alginate fermentation. Cells showed vigorous growth in first 48 h, suggesting alginate is favored by Alg1, and a total of 7.6 g/L alginate was consumed after 108 h (Fig. 1c). Totally, 2.7 g/L ethanol and 3 g/L acetic acid were produced, with an ethanol-to-acetate ratio of 0.9:1. It can be concluded that *D. phaphyphila* Alg1 could successfully convert alginate into ethanol and acetate, and its products contained more acetic acid than those from glucose and mannitol. It is worth to mention that no natural strains have been reported to ferment alginate, which might attribute to the fact that alginate needs a complex system for decomposition, and the resulting uronic acid is not easily converted by strains without ED pathway. This indicates that *D. phaphyphila* Alg1 has good potential for ethanol production from seaweed compared to other reported strains [1, 11, 21, 32].

**Fig. 2:**
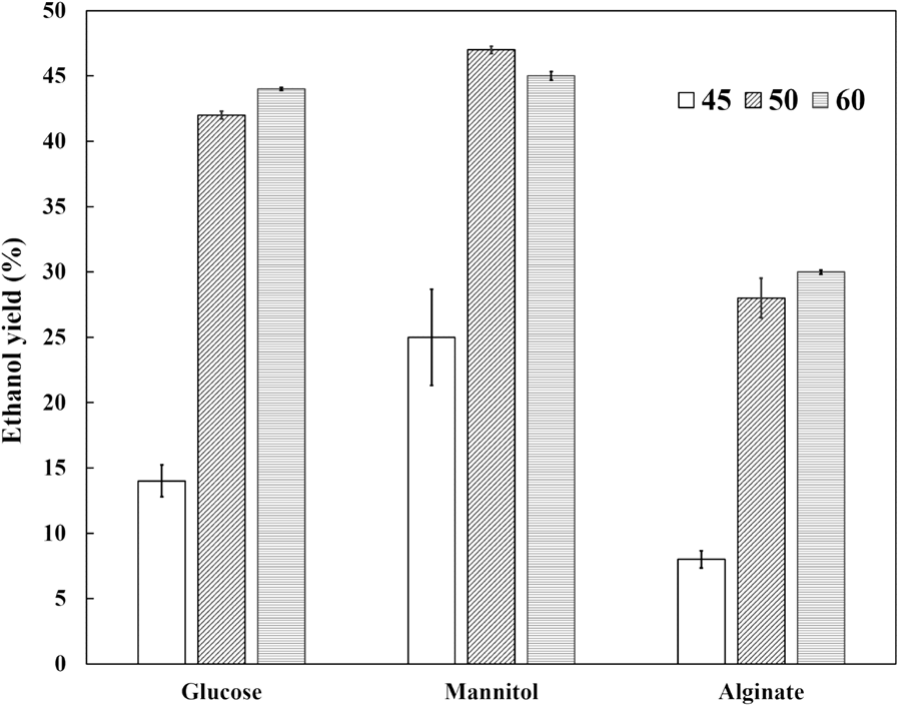
Ethanol yields of *D. phaphyphila* Alg1 from glucose, mannitol, and alginate under different temperatures

One of the most important characteristics shared by Alg1 is that it is a thermophilic strain producing high concentration of ethanol, which was very rare in the previous reports. The effects of cultivation temperature on ethanol yields were evaluated in this work. The growth temperatures of *D. phaphyphila* Alg1 were from 45 to 65 °C based on the previous work [27]. Accordingly, three temperatures (45, 50, and 60 °C) were chosen to estimate the effects on ethanol yields. As shown in Fig. 2, *D. phaphyphila* Alg1 showed higher ethanol productivities at both 50 and 60 °C than at 45 °C. The highest ethanol yields for glucose and alginate were at 60 °C, which were 0.44 g/g-glucose and 0.3 g/g-alginate. The ethanol yields for mannitol did not show significant difference at 50 and 60 °C, which were 0.44 g/g-mannitol and 0.42 g/g-mannitol, respectively. It can be concluded that Alg1 showed better substrate conversion rates between 50 and 60 °C. Thus, *D. phaphyphila* Alg1 turned out to be a unique thermophilic ethanologenic bacterial strain with high ethanol yields from glucose, mannitol, and alginate.

### Genome analysis of ethanol synthesis pathway and redox balance analysis

**Fig. 3:**
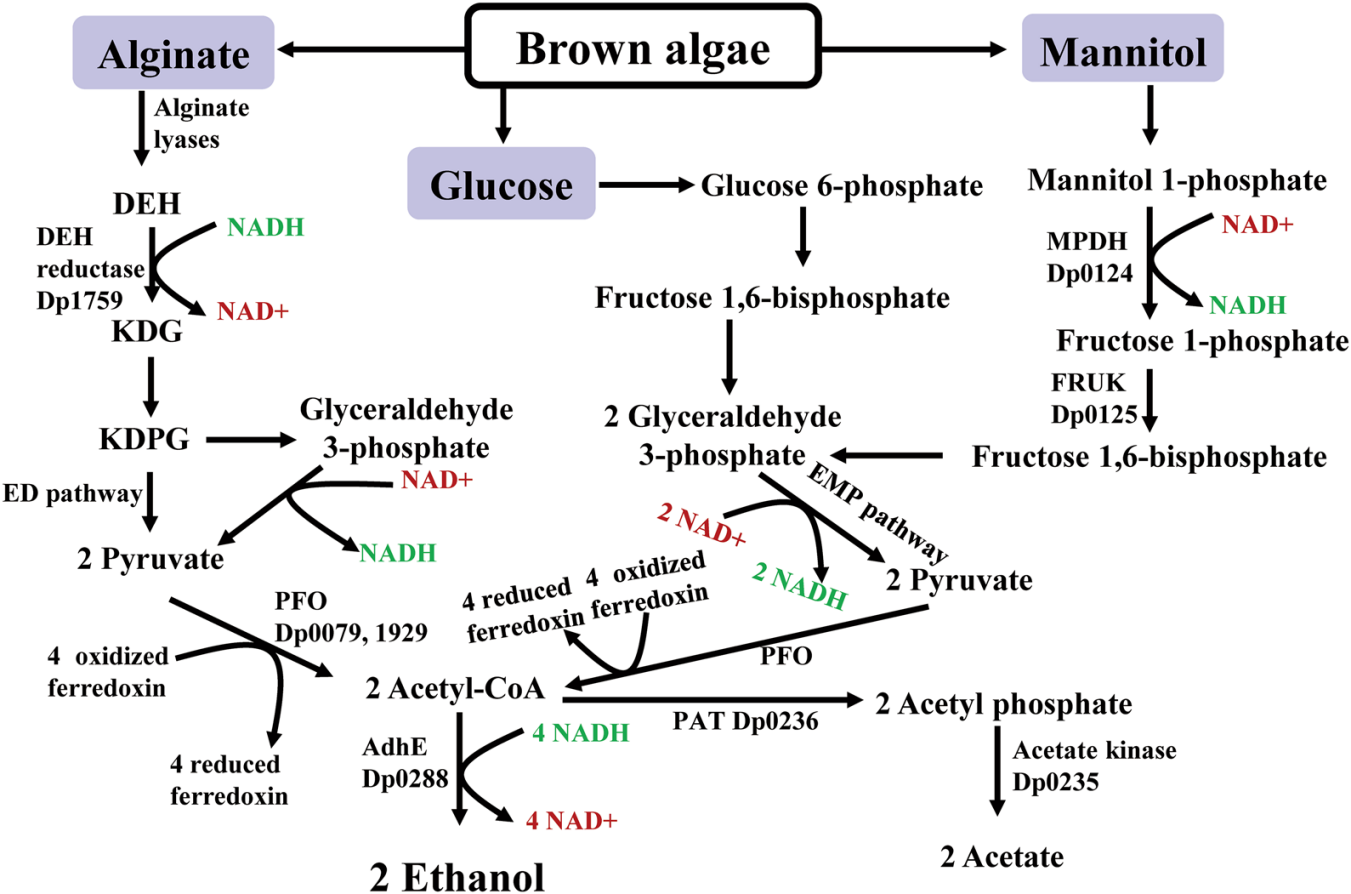
Genome analysis of the metabolic pathways and redox balance of ethanol synthesis in *D. phaphyphila* Alg1. *EMP* Embden–Meyerhof–Parnas; *ED* Entner–Doudoroff; *AdhE* bifunctional alcohol and aldehyde dehydrogenase; *PFO* pyruvate ferredoxin oxidoreductase; *PAT* phosphate acetyltransferase; *MPDH* mannitol-1-phosphate dehydrogenase; *FRUK* 1-phosphofructokinase

Ethanol synthesis from the three major components of brown algae-glucose, mannitol, and alginate was analyzed from genome information. As shown in Fig. 3, the ethanologenesis starts with the formation of pyruvate through EMP (Embden–Meyerhof–Parnas) pathway and ED pathway, and pyruvate is then converted to acetyl-CoA by pyruvate:ferredoxin oxidoreductase (PFO) encoded by Dp0079 and Dp1929. Lastly, ethanol was produced from acetyl-CoA by a bifunctional alcohol and aldehyde dehydrogenase (AdhE, Dp0288) which shared 67 % of identity with the AdhE of *Thermoanaerobacterium saccharolyticum* with consumption of 2 NADH [33], and acetate was produced by a phosphate acetyltransferase (PAT, Dp0236) and an acetate kinase (Dp0235) without consumption of NADH (Fig. 3). Through the genome analysis, it can be concluded that *D. phaphyphila* Alg1 utilizes a pathway containing PFO to assimilate pyruvate. This pathway, which was also found in a famous engineered thermophilic bacterium *Thermoanaerobacterium saccharolyticum* with high ethanol titer, is different from that in previously described microbes with homoethanol fermentation [34]. According to the metabolic pathway, to produce 2 ethanol, the net consumptions of NADH for glucose and mannitol were 2 NADH and 1 NADH, respectively (Fig. 3). To produce 2 acetate, 2 NADH and 3 NADH were generated in the catabolism of glucose and mannitol, respectively. In the case of alginate assimilation, the feasible pathway of producing ethanol from this polysaccharide was rather rare in microorganisms. To decompose alginate, it was firstly depolymerized into DEH, and then NADH-dependent DEH reductases (DehR) were employed to produce KDG; lastly, KDG was fed into the ED pathway to produce pyruvate and glyceraldehyde-3-phosphate (G3P) by 2-keto-3-deoxyglucononate kinase (KdgK) and KDPG aldolase (KdpgA), which has been discussed in our previous work [27]. During this process, 1 NADH was consumed by DehR, and thus would cause the deficiency of reducing equivalents. To generate 2 ethanol, 4 NADH were consumed, while NADH maintained a balance in generating 2 acetate. The vigorous growth in mannitol, glucose, and alginate under obligate anaerobic condition indicated that *D. phaphyphila* Alg1 could well balance the redox level.

To figure out the redox balance in metabolism of mannitol, glucose, alginate, and their mixture, the NAD/NADH ratio in the cells was analyzed (Table 1). In accordance with the results of genome analysis, the low NAD/NADH ratio (0.63 ± 0.15) in mannitol metabolism indicated that excessive reducing equivalents were produced, while the high NAD/NADH ration (7.93 ± 1.07) in alginate assimilation indicated a deficiency of reducing equivalents (Table 2). Alg1 showed a middle NAD/NADH ratio (3.48 ± 0.57) when fermenting the mixture of alginate, mannitol, and glucose (5:8:1) simulating the main components of brown algae. Through these results, it can be concluded that the different oxidoreduction potentials in substrates of glucose, mannitol, and alginate finally affected the metabolic flux into ethanol pathway. Ethanol production highly consums reducing equivalents. On the one hand, the lack of NADH could explain a low ethanol-to-acetate ratio in alginate degradation, which means production of acetic acid was a direct response to deficiency of reducing power. On the other hand, the consumption of excess reducing equivalents is also important in balancing the redox potential. The redox balance is hard to control especially in engineered strains. For example, *Sphingomonas* sp. A1 was engineered to produce ethanol from alginate, and oxygen supply should be strictly controlled to maintain the redox balance between energy supplement and ethanol production [6]. Even under precise control, *Sphingomonas* sp. strain A1 could only reach an ethanol yield of 0.25 g/g-alginate which was lower than that of strain Alg1 (0.3 g/g-alginate).

**Table 1:** The redox states of *D. phaphyphila* Alg1 in fermenting different sugars of brown algae

	NAD + NADH	NAD	NAD/NADH
Mannitol	1.77 ± 0.12	0.60 ± 0.16	0.63 ± 0.15
Glucose	1.75 ± 0.70	1.17 ± 0.42	2.51 ± 0.51
Alginate	2.70 ± 0.52	2.45 ± 0. 44	7.93 ± 1.07
Sugar mixture^a^	2.18 ± 0.15	1.69 ± 0.53	3.48 ± 0.57

^a^Sugar mixture contained alginate, mannitol and glucose with a ratio of 5:8:1

**Table 2:** Microorganisms that convert brown seaweed biomass to ethanol

Microorganism	Characteristics	Major sugars utilized	Pretreatment	References
*D. phaphyphila* Alg1	Thermophilic Nat	Mannitol, alginate, laminarin	Un-pretreatment	This study
*Zymobacter palmae*	Nat	Mannitol, laminarin	Thermal acid hydrolysis	[8, 39]
*Pichia angophorae*	Nat	Monosaccharide	Thermal acid hydrolysis	[32]
*Saccharomyces cerevisiae*	Nat	Glucose	Laminarinase	[17]
*Escherichia coli* BAL1611	Eng	Mannitol, alginate, glucose	Un-pretreatment	[23]
*Escherichia coli* KO11	Eng	Glucose, mannitol	Acid and hydrolytic enzymes	[11]
*Sphingomonas* sp. A1	Eng	Alginate	N/A	[6]
*Saccharomyces cerevisiae* BAL3215	Eng	Mannitol, glucose, DEH	N/A	[20]

*Nat* stands for natural strains, *Eng* stands for engineered strains, and *N/A* means not applicable

The different oxidoreduction potentials of glucose, mannitol, and alginate make the bioconversion of brown algae very difficult. The deficiency of reducing equivalents in alginate metabolism, and the highly reducing equivalents consumption during ethanol formation can easily cause serious redox imbalance. No natural microorganisms have been reported to be capable of converting alginate to ethanol as far as we know. As mentioned above, mannitol is also hard to be converted to ethanol under anaerobic condition in *Z. palmae*, *Enterobacter* sp. JMP3, and some yeast species. Mannitol fermentation would produce excess electrons in contrast to alginate. Under anaerobic condition, these excess reducing equivalents could not be neutralized by oxygen, especially when the microorganisms lack transhydrogenase (an enzyme converting catabolic reducing equivalent NADH to anabolic reducing equivalent NADPH) [8, 35]. However, the redox problem was well solved in *D. phaphyphila* by integrating two complementary pathways which were hard to proceed individually to reach equilibrium. These results provide insights into mechanisms of redox balancing in fermentation of brown algae, and will assist future metabolic engineering efforts on *D. phaphyphila*.

### Ethanol fermentation of brown algae by *D. phaphyphila* Alg1

**Fig. 4:**
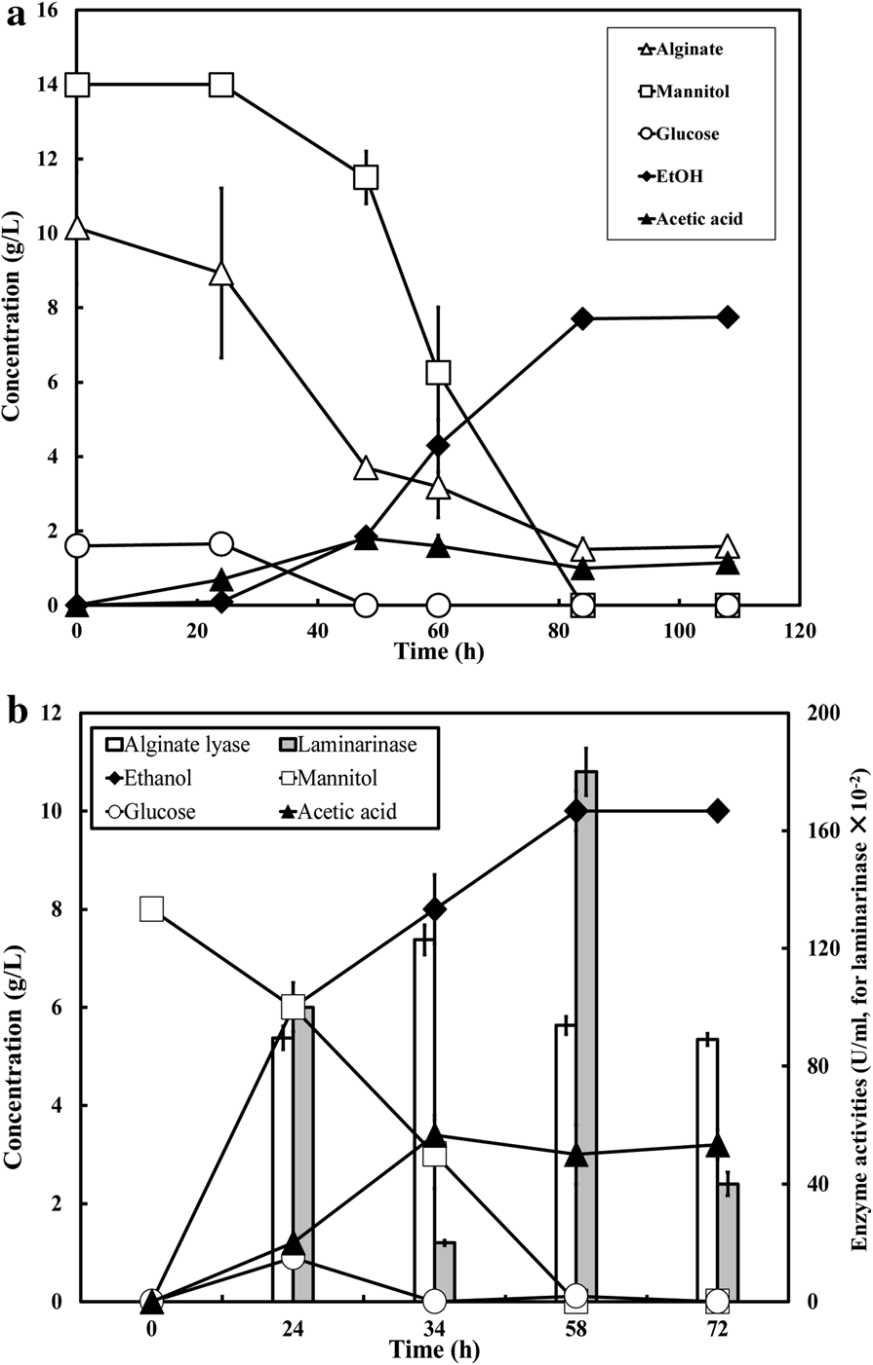
Fermentation properties of *D. phaphyphila* Alg1 using mix sugars (alginate:mannitol:glucose = 5:8:1) simulating main components of brown algae (**a**) and kelp powder (**b**) as substrates

An artificial mixed sugar simulating the main components of brown algae with a ratio of alginate:mannitol:glucose = 5:8:1 (3 % total sugar) was used to evaluate the fermentation properties of Alg1, which would help us to have a comprehensive understanding of the fermentation process. As shown in Fig. 4a, a total of 7.8 g/L ethanol and 1.2 g/L acetic acid were produced with a consumption of 24.2 g/L total sugars. The ethanol-to-acetate ratio and ethanol yield were calculated to be 6.5:1 and 0.32 g/g-total sugar, respectively. The contents of alginate, mannitol, and glucose decreased quickly after 24 h fermentation, and a simultaneous consumption of them was observed during 24–48 h. It can be concluded that *D. phaphyphila* Alg1 could simultaneously saccharify and ferment the three main components of brown algae, and although these components have different oxidoreduction potentials, reducing equivalents are well balanced and metabolic flux is directed into ethanol production in this strain.

We further used 5 % (w/v) kelp powder (dry weight 48 g) to evaluate the ethanol production ability of *D. phaphyphila* Alg1 in 1-L bioreactors. Through 72 h fermentation, 10 g/L of ethanol and 3.2 g/L of acetic acid were produced (Fig. 4b). Mannitol was released to the medium immediately after the kelp powder was added with a concentration of 8 g/L. Initially, glucose was undetectable, while an increasing amount of glucose was observed after the initial cell growth, suggesting that the secreted hydrolases caused the degradation of laminarin and the release of glucose. This hypothesis process was confirmed in the enzyme activity assay (Fig. 4b). At the end of fermentation, a total of 40 g/L kelp powder was consumed, corresponding to an ethanol yield of 0.25 g/g-kelp.

As an emerging feedstock for liquid biofuel production, brown algae bioconversion has been extensively studied. Some of the studies on converting brown seaweed to ethanol are listed in Table 2. Both natural and engineered strains were employed for bioconversion. For most studies, a multiple-step process with acid and/or enzymatic pretreatments before fermentation was used. For example, Kim et al. used *E. coli* KO11 to ferment *S. japonica* with acid pretreatment and enzymatic hydrolysis, and the study resulted in an ethanol yield of 0.4 g/g of sugars [11]. In spite of the high ethanol yield of strain KO11, it still lacks the ability of biomass saccharification. The inability of alginate and mannitol fermentation by natural ethanologenic microorganisms can be concluded from Table 2. The low ethanol yield of yeast was likely due to its inability in utilizing mannitol. The molecular basis of this inability of mannitol assimilation in *S. cerevisiae* was illustrated very recently [36]. Some specific engineered strains have displayed excellent performance in assimilating unpretreated brown algae. The ethanol yield of *E. coli* strain BAL1611 was 0.29 g/g-seaweed at 25 °C [23]. Compared with these previous studies, there are two impressive characteristics of *D. phaphyphila* Alg1 in brown algae bioconversion. Firstly, *D. phaphyphila* Alg1 is an obligate anaerobic and thermophilic bacterium, which can ferment brown algae under elevated temperatures up to 60 °C. Thermophilic bacteria could offer the advantages for ethanol production such as low contamination risk [37]. Secondly, *D. phaphyphila* Alg1 can directly assimilate unpretreated brown algae with high ethanol conversion rate, and can simultaneously ferment the mannitol, laminarin, and alginate, which are highly advantageous over most microorganisms. In a word, *D. phaphyphila* Alg1 possesses not only extracellular polymer-degrading enzymes such as alginase and laminarinase for saccharification but also good ethanol production ability, which would potentially provide it advantages in bioconversion process. Similar to the consolidated bioprocessing (CBP) process in the conversion of lignocellulose into biofuel in one step, the direct conversion of brown algae to ethanol has potential to lower the cost of biomass processing due to elimination of operating and capital costs associated with dedicated enzyme production and/or thermal acid hydrolysis, and a more effective biomass solubilization could be achieved [38]. More than that, the ethanol yields of these strains used in the two-step methods were lower than that of *D. phaphyphila* because of their inability of utilizing monomers of alginate and/or mannitol.

### Biodegradation characteristics of brown alga cell wall

**Fig. 5:**
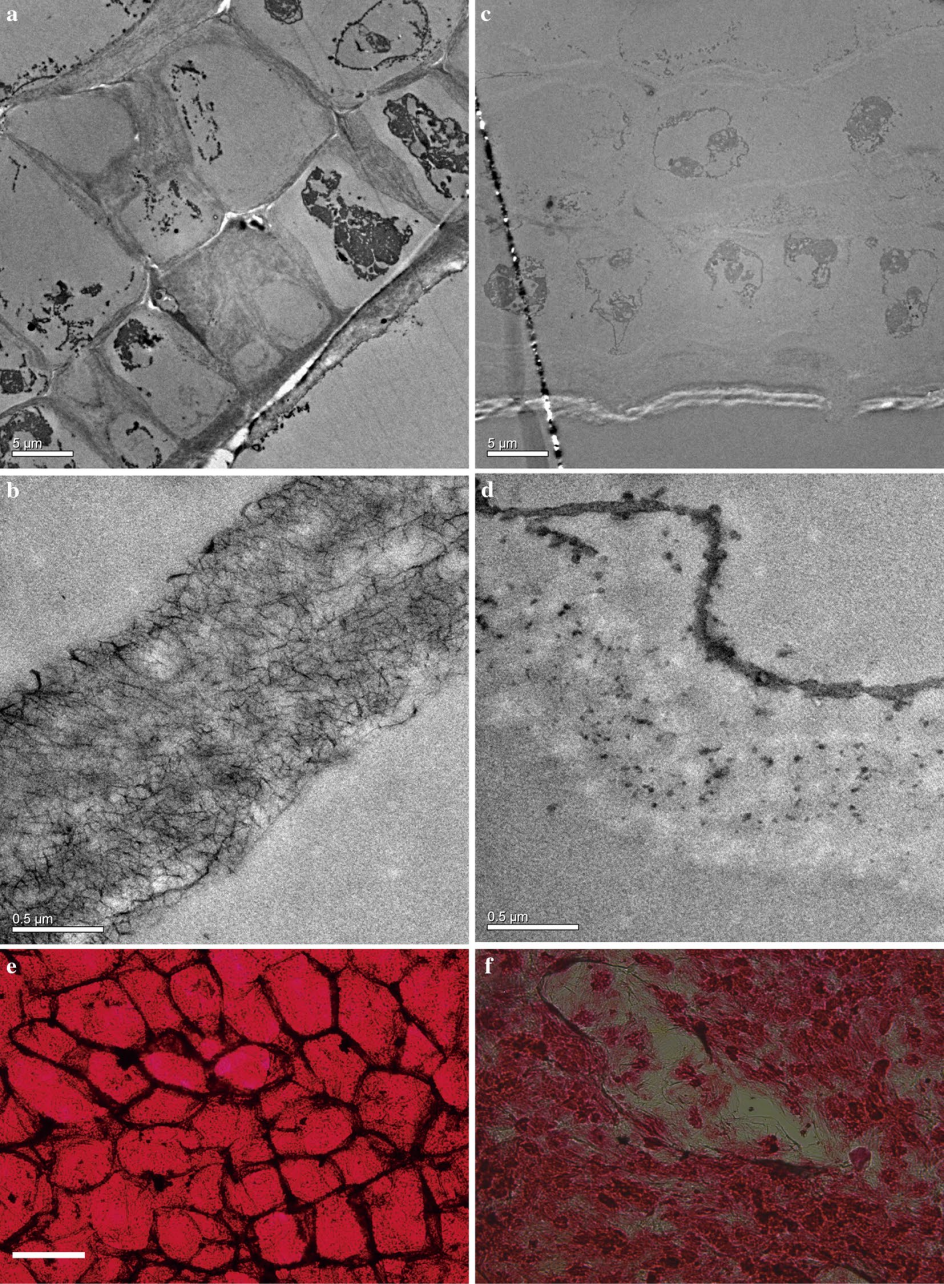
Transmission electron microscope (TEM) and optical microscope images of brown algae samples, unfermented brown algae (**a**, **b**, **e**), and brown algae after fermentation (**c**, **d**, **f**). **e**, **f** stained with azaleine, and *scale bars* 100 μm

To elucidate the biodegradation characteristics of *D. phaphyphila* Alg1 on brown algae, strips of *S. japonica* was used as substrate. The morphological changes induced by incubation with *D. phaphyphila* Alg1 were examined by both transmission electron microscope (TEM) and optical microscope to provide direct insight into the structure modification of the biomass. Before fermentation, a high electron density of the cell walls observed by TEM indicated the cell integrity (Fig. 5a, c). Similarly, ordered cell walls could be easily recognized by optical microscope after being stained with azaleine (Fig. 5e), and the cell showed high mechanical strength. After 120 h fermentation by *D. phaphyphila* Alg1, the morphology of residual brown algae changed dramatically. The electron density of the cell walls became very light (Fig. 5b, d). From optical microscopy, the initial ordered cell structures were destroyed, and were subsequently presented as collapsed and incomplete cell structures (Fig. 5f). Obviously, the cell wall components sustaining cell morphology which are mainly composed of alginate and glucan were largely degraded during fermentation. In addition to microscopic observation, the activities of corresponding depolymerizing enzymes during fermentation of brown seaweed were analyzed. As shown in Fig. 4b, alginate lyase and laminarinase activities were detected. The alginate lyase activity increased rapidly before 24 h and reached a maximum level after 34 h of incubation at 123 U/mL. This activity maintained at a high level during fermentation, indicating Alg1 has an evolved thermostable alginolytic system which has been proved in previous work [27]. The laminarinase activity was consistent with the appearance of glucose. High activity was detected at 24 h, and the highest concentration of glucose was achieved at the same time, which meant that laminarinase caused the release of glucose from laminarin. The high concentration of glucose might cause a negative feedback inhibition of the laminarinase gene expression. In the next 10 h, no more laminarinases were produced, and the formerly synthesized laminarinases gradually lost their activities. Therefore, the laminarinase activity became weaker at 34 h. At the same time, glucose was consumed immediately, and was exhausted at 34 h. The inhibition of laminarinase gene was relieved accordingly, and more laminarinases were synthesized to produce more glucose. The result is that laminarinase activity undergoing a short decrease achieved the maximum activities of 1.8 U/mL at 58 h (Fig. 4b). However, only small amount of glucose was detected at this time for most of laminarin had been hydrolyzed. These results further confirmed the decomposition of unpretreated brown algae cell walls and their polysaccharides by strain Alg1.

## Conclusions

*Defluviitalea phaphyphila* Alg1 showed remarkable ethanol production ability in assimilating brown algae. High ethanol yields of 0.44, 0.47, and 0.3 g/g of sugars were obtained by fermenting glucose, mannitol, and alginate at 60 °C, respectively. An ethanol yield of 0.25 g/g-biomass was obtained in fermenting unpretreated kelp. Genome and redox analysis revealed a well evolved redox balance system in Alg1 in fermenting brown algae. The elucidation of the redox balances of substrates with different oxidoreduction potentials would help us increase ethanol productivity. This novel bacterium provides a potential gene-manipulable platform for high-value chemical production from brown algae at elevated temperature.

## Abbreviations

PTS: phosphotransferase
MPDH: mannitol-1-phosphate dehydrogenase
NADPH: reduced nicotinamide adenine dinucleotide phosphate
NADH: reduced nicotinamide adenine dinucleotide
DEH: 4-deoxy-L-erythro-5-hexoseulose uronic acid
KDG: 2-keto-3-deoxy-d-gluconate
KDPG: 2-keto-3-deoxy-6-phosphogluconate
ED: Entner–Doudoroff
HPLC: high-performance liquid chromatography
DNS: dinitrosalicylic
EMP pathway: Embden–Meyerhof–Parnas pathway
PFO: pyruvate:ferredoxin oxidoreductase
AdhE: bifunctional alcohol and aldehyde dehydrogenase
PAT: phosphate acetyltransferase
FRUK: 1-phosphofructokinase
DehR: 4-deoxy-L-erythro-5-hexoseulose uronic acid reductases
G3P: glyceraldehyde-3-phosphate
KdgK: 2-keto-3-deoxyglucononate kinase
KdpgA: 2-keto-3-deoxy-6-phosphogluconate aldolase
TEM: transmission electron microscope
